# The skin microbiome on healthy and inflammatory altered canine skin determined by next generation sequencing

**DOI:** 10.3389/fmicb.2025.1528747

**Published:** 2025-02-27

**Authors:** Lucia Štempelová, Lenka Micenková, Petr Andrla, Viola Strompfová

**Affiliations:** ^1^Centre of Biosciences of the Slovak Academy of Sciences, Institute of Animal Physiology, Košice, Slovakia; ^2^Department of Experimental Biology, Faculty of Science, Masaryk University, Brno, Czechia; ^3^RECETOX, Faculty of Science, Masaryk University, Brno, Czechia; ^4^Department of Biology, Faculty of Medicine, Masaryk University, Brno, Czechia

**Keywords:** skin, dog, microbiota, 16S rRNA gene, streptococci, staphylococci

## Abstract

**Introduction:**

Human and animal skin is colonized by a complex microbial population. An imbalance of these microorganisms is often associated with dermatological diseases.

**Methods:**

The aim of this work was to describe the skin bacterial microbiota composition of healthy dogs and dogs with inflammatory skin lesions. Genomic DNA was sequenced using primers that target the V4 region of the bacterial 16S rRNA gene. Superficial skin swabs were collected from eight body areas of six healthy dogs (*n* = 48) and directly from inflammatory altered canine skin (*n* = 16).

**Results:**

The skin of healthy dogs was predominantly colonized by phylum Bacillota (34.4 ± 27.2%), followed by Actinomycetota (32.2 ± 20.3%), Pseudomonadota (16.4 ± 12.2%), and Bacteroidota (8.7 ± 11.6%). At the level of genera, *Streptococcus* spp. (19.4 ± 26.1%) was the most abundant genus across all samples collected from healthy skin, followed by *Curtobacterium* (5.4 ± 12.1%), *Bacteroides* (5.2 ± 11.1%) and *Corynebacterium_*1 (4.3 ± 13.2%). More specifically, *Streptococcus* spp. was the most abundant on the chin (49.0 ± 35.5%), nose (37.9 ± 32.1%), perianal region (21.1 ± 28.2%), abdomen (11.0 ± 12.8%), dorsal back (12.4 ± 10.3%) and interdigital area (5.5 ± 2.2%). *Curtobacterium* spp. was predominant on inner pinna (17.8 ± 24.8%) and axilla (6.7 ± 10.8%). Alpha diversity analysis (Shannon index) showed maximum on interdigital area but minimum on a chin (*p*-value: 0.0416). Beta diversity analysis showed clustering across samples from the individual skin sites but also across samples collected from individual dogs. *Staphylococcus* spp. was the most abundant genus in 12/16 samples collected from inflammatory skin. In addition, a lower bacterial diversity was observed in samples from skin lesions compared to samples from healthy canine skin.

**Discussion:**

The results confirm the fact that the microbiome of healthy skin is very diverse. Compared to other studies, streptococci predominated on healthy canine skin. Shannon index showed only minor differences in diversity between different parts of canine skin. Results of beta-diversity showed the fact that the main force driving the skin microbiota composition is the individual, followed by the skin site. On the area of skin lesions, dysbiosis was observed with a significant predominance of staphylococci.

## 1 Introduction

The skin is the largest epithelial interface isolating organism from the outside environment and its surface is colonized by a diverse community of bacteria, fungi, and viruses (Gallo, 2017). The skin commensals have many important functions: promote defence and immune responses, inhibit colonization and infection by opportunistic or pathogenic organisms and promote tissue repair and barrier functions (Flowers and Grice, 2020). The microorganisms inhabiting the skin must overcome numerous challenges that typically prevent microbial growth, including low pH, osmotic pressure, and low nutrient availability (Swaney et al., 2023). With the recent advent of molecular biology and next generation sequencing (NGS) as tools for microbiological identification, knowledge about the skin microbiota has grown exponentially.

It is currently known that human skin microbiome varies topographically due to different physiology of the skin site, with specific bacteria being associated with moist, dry and sebaceous microenvironments. However, such dividing of the canine is not feasible because of several anatomic differences between human and canine skin. Thick fur, apocrine glands and sebaceous glands are more evenly distributed throughout the body (Miller et al., 2012; Hoffmann, 2017). Close contact with external environment may also modulate colonization by the skin microbiota (Hoffmann et al., 2014). It is important to study taxonomic profile of the canine skin that gives clear insights into the structure of the skin microbiome and enables us to find out which taxa are responsible for the health of the skin also by comparing with microbiome of diseased skin. It is necessary to achieve overview on composition worldwide to recognize transient environmental from resident bacterial taxa in dogs.

The homeostasis of the skin microbiome plays a key role in protection against skin disorders. Its disruption by different factors manifesting by (1) loss of beneficial bacteria, (2) excessive growth of potentially pathogenic bacterial species and by (3) reduction in community diversity leads to a state of dysbiosis associated with an altered immune response promoting the development of skin diseases (Boxberger et al., 2021; De Pessemier et al., 2021). Most of the available data regarding alterations of the skin microbiome in diseases come from human studies despite the fact that skin diseases are very frequent especially in dogs (Nationwide, 2019). Common skin diseases in dogs include atopic dermatitis, allergies, skin infections, pyoderma, otitis externa or fungal infections (Hill et al., 2006). In these diseases, recurrence is likely unless the underlying disease, dysbiosis, or structural abnormality is identified and managed (Loeffler and Lloyd, 2018). Knowledge on the animal microbiota is important also because of sharing microbiota between animals and humans. Interestingly, Song et al. (2013) found that skin microbiota of adults who own dogs are more similar to other dog owners than non-dog owning adults.

Although there has been a substantial amount of research into the composition of the canine microbiome there is still much to discover. The aim of this study was firstly to provide information on the taxonomic composition and diversity of bacterial community inhabiting different areas of the healthy canine skin including mucosal surface. The second aim was to compare the skin microbiome of healthy dogs with that of dogs with skin lesions altered by inflammation. The study adds to a growing collection of research that try to better understand the microbial composition differences of the healthy vs. unhealthy skin in dogs what it is essential tool for creation larger confirmatory studies but also for the further development of new therapeutic approaches.

## 2 Materials and methods

### 2.1 Collection of samples

Superficial skin swabs were obtained from healthy dogs and dogs with inflammatory skin conditions in cooperation with veterinary clinics (Small Animal Clinic, University of Veterinary Medicine and Pharmacy, Košice, Slovakia). The skin swabs were collected (December 2019–June 2020) from eight body areas (left inner pinna, nasal area, chin, dorsal back, abdomen, left axilla, interdigital area of the left fore paw and perianal area) of six healthy dogs (*n* = 48) and from lesions with inflammatory skin conditions from 16 ill dogs (*n* = 16). We aimed at acute inflammatory lesions regardless of etiology characterized by redness, with pain and itching to different degrees (average skin lesion score 2.2 ± 1.3, skin lesion scoring system from 0-no lesions to 5-many very large, deep, and red lesions; pruritus score 2.4 ± 1.1; 0-no pruritus, 5-severe) possibly with alopecia before treatment started. The age of healthy dogs was 7.8 ± 4.7 while 5.5 ± 3.0 in ill dogs. The proportion of gender was similar (1:1). All dogs involved in the study came from area of Košice (Slovakia). All healthy dogs had no clinical symptoms of the disease and had no history of antimicrobial exposure within the preceding 3 months. All dogs were kept indoors, but they were taken out two to three times a day. The healthy dogs were fed with commercial dry dog food (Brit, Vafo, Czech Republic) and cooked meat with vegetables. The ill dogs were also fed with a combination of fresh and kibble dog food twice a day. Sample collection from healthy dogs was performed in a room of their home unit while ill dogs were sampled in the ambulance of the University of Veterinary Medicine and Pharmacy. Samples were obtained by firmly rubbing each area 20 times (10 strokes per swab side) using FLOQSwabs (Copan, Italy) soaked in sterile SCF-1 solution (50 mM Tris buffer, 1 mM EDTA and 0.5% Tween-20). The collection tube was filled with 2 mL of the stabilization solution DNA/RNA Shield R1100-250 (Zymo Research, Irvine, United States). The samples were than frozen at -80°C until processing. To prevent cross-contamination, the person performing sampling wore a pair of sterile gloves for each individual area. Dog owners completed the questionnaire (name, breed, sex, age, locality) and they agreed with sampling. In dog patients, the results were supplemented with information regarding the size of the lesions and pruritus score (Supplementary Table 1 in Supplementary material S1).

### 2.2 DNA isolation and sequencing

DNA isolation of 64 skin swabs was performed using a PowerLyzer^®^ PowerSoil^®^ DNA Isolation kit (QIAGEN, Germany), according to the manufacturer's protocol protocol. Isolated DNA was used as a template in PCR reactions targeting the hypervariable V4 region (EMP 515-806) of the bacterial 16S rRNA gene according to the 16S Metagenomic Sequencing Library Preparation protocol (Illumina, San Diego, CA) (Supplementary Table 2 in Supplementary material S1). Sequencing was performed using MiSeq Reagent Kits v2 on a MiSeq 2000 sequencer according to the manufacturer's instructions instructions (Illumina, United States).

The sequencing reads were processed by an in-house tool written in Python 3. Reads were demultiplexed into individual samples based on unique 7–9 bp tag sequences within the first 30 nucleotides. After demultiplexing, tags and an additional 30 nucleotides were removed to eliminate adapter sequences. Low-quality ends were trimmed based on a phred score threshold, with the trimming length determined separately for forward and reverse reads. Pairs with ambiguous nucleotides or reads shorter than the calculated length threshold were discarded. Next, forward and reverse reads were denoised using the DADA2 amplicon denoising R package. This was done in order to cope with the sequencing and PCR-derived error. For denoising, no truncation was applied, reads with more than 2 expected errors were discarded. Following denoising, the forward and reverse reads were joined using the fastq-join read joining utility. In order to be joined, reads in pair had to have an overlap of at least 20 bp with no mismatches allowed. Pairs in which this was not the case were discarded. Chimeric sequences were removed using the DADA2 algorithm (Aronesty, 2011; Callahan et al., 2016). The taxonomy was determined using the usearch-consensus algorithm from the microbiome analysis toolkit QIIME (v 1.9.1) (Caporaso et al., 2010). For each input sequence, the three closest organisms were found in the Silva v. 123 reference database (Quast et al., 2013). Their taxonomies were combined into the final taxonomic assignment using the least common ancestor (LCA) algorithm. Taxonomic names of bacterial phyla obtained from Silva database were corrected according to the publication by Oren and Garrity (2021). Sterile swabs were processed as negative workflow controls to monitor for contamination introduced during sample handling and all laboratory processing.

### 2.3 Data analysis

Subsequent analysis and visualization were performed on the online MicrobiomeAnalyst server (Chong et al., 2020). MicrobiomeAnalyst comprises four modules, and we used the Marker Data Profiling (MDP) module that is designed for analysis of 16S rRNA marker gene survey data. The samples with less than 2,000 read counts per sample were excluded from further analysis. The analysis yielded 2,193,007 total reads distributed across 58 samples with a minimum and maximum number of reads per sample of 2,141 and 127,761, respectively. An average of 37,810 read counts were obtained per sample. Before downstream analysis of alpha and beta diversity, the ASV table was filtered to remove spurious ASVs (10% of prevalence in samples). Core microbiome, alpha and beta diversity analyses were also performed in the Microbiome Analyst platform (Chong et al., 2020). Core microbiome analysis refers to the study of the consistent microbial taxa found in group of samples. Alpha diversity analysis assesses the diversity within a sample. Alpha diversity parameter Shannon was used for comparison of different areas of healthy skin and also for comparison healthy and diseased canine skin. All comparisons were done through a *t*-test/ANOVA (analysis of variance) at the level of genera.

We also performed beta diversity analysis which assesses the similarities among samples of the same community. It provides a measure of the distance or dissimilarity between each sample pair. Beta diversity is calculated for every pair of samples to generate a distance or dissimilarity matrix, reflecting the dissimilarity between certain samples. PCoA plots were created by applying the PERMANOVA algorithm on the Bray Curtis distances of bacterial genera. Also, statistical method pairwise PERMANOVA was applied to compare results between two groups of samples. Resulting *p*-values were corrected for multiple comparisons using false discovery rate (FDR) method (Benjamini–Hochberg). A significance threshold of *q* < 0.05 was used to identify differentially abundant bacteria. An *R* value near 1 means that there is dissimilarity between the groups, while an *R* value near 0 indicates no significant dissimilarity between the groups.

The potential function of skin bacterial communities was assessed using 16S rRNA sequencing data and the predictive software Phylogenetic Investigation of Communities by Reconstruction of Unobserved States (PICRUSt) (Langille et al., 2013). The Kyoto Encyclopedia of Genes and Genomes (KEGG) hierarchy was used to infer the functional content of genes (Kanehisa and Goto, 2000).

## 3 Results

### 3.1 Bacterial composition of the skin of healthy dogs

A total of 33 bacterial phyla were identified across skin samples taken from healthy dogs. In general, the phylum Bacillota (34.4 ± 27.2%) was predominant and was followed by Actinomycetota (32.2 ± 20.3%), Pseudomonadota (16.4 ± 12.2%), and Bacteroidota (8.7 ± 11.6%) phyla. The phylum Bacillota dominated only in the perianal area (60.6 ± 29.6%), on the chin (56.4 ± 30.3%) and in the nasal area (50.8 ± 29.3%). In contrast, the phylum Actinomycetota dominated on inner pinna (56.3 ± 25.4%), dorsal back (34.7 ± 8.8%), axilla (39.6 ± 10.2%), abdomen (37.6 ± 8.3%), and interdigital area (37.1 ± 9.2%).

**Figure 1:**
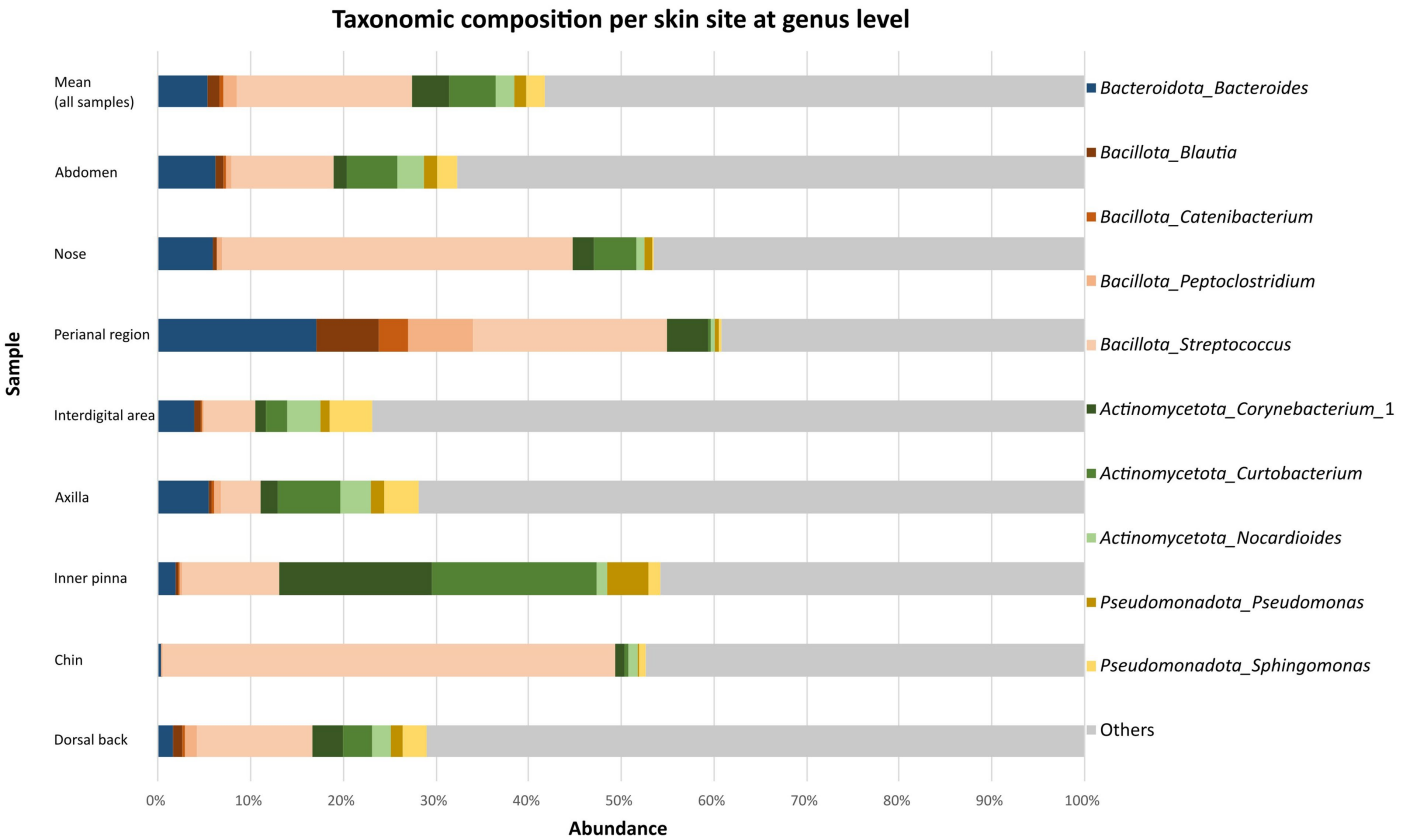
Taxonomic composition per skin site at genus level. Bar graphs show the mean relative abundances of top 10 the most abundant bacterial genera at each body site (abdomen, nose, perianal region, interdigital area, axilla, inner pinna, chin, dorsal back).

The taxonomic classification at the genus level resulted in 533 classified genera. Genus *Streptococcus* considerably predominated (19.4 ± 26.1%) and was followed by *Curtobacterium* (5.4 ± 12.1%), *Bacteroides* (5.2 ± 11.1%) and *Corynebacterium_*1 (4.3 ± 13.2%). *Streptococcus* spp. was the most abundant on the chin (49.0 ± 35.5%), nose (37.9 ± 32.1%), perianal region (21.1 ± 28.2%), abdomen (11.0 ± 12.8%), dorsal back (12.4 ± 10.3%) and interdigital area (5.5 ± 2.2%). The samples from the inner pinna and axilla were slightly different. On the inner surface of pinna was the most abundant genus *Curtobacterium* (17.8 ± 24.8%). Genera like *Corynebacterium_*1 (16.4 ± 31.1%), *Streptococcus* (10.5 ± 13.7%), *Pseudomonas* (4.4 ± 9.6%) and *Bacteroides* (1.9 ± 1.5%) were found in lower abundance on this site. On the axilla, genus *Curtobacterium* (6.7 ± 10.8%) was also the most abundant genus according mean relative abundance in dogs. *Bacteroides* spp. (5.4 ± 8.5%), *Streptococcus* spp. (4.4 ± 3.3%) and *Sphingomonas* (3.7 ± 2.3%) were detected in approximately similar abundance in this area (Figure 1). Mean relative abundances of other taxa on different parts of the body are summarized in Supplementary Tables 3–5 in Supplementary material S1.

A core microbiome analysis was also performed to check the prevalence (presence in the number of samples) of these all detected genera. Maximum prevalence was shown by *Streptococcus* (0.83) followed by *Corynebacterium_*1 (0.64) and *Nocardioides* (0.55). The rest had prevalence below 0.55 (Supplementary Table 6 in Supplementary material S1).

Statistical analysis was done to compare abundances of bacterial phyla and genera among individual skin sites. When the phyla with the highest mean relative abundance were considered (top four), significant differences corrected using FDR were observed only in Bacillota (inner pinna vs. perianal region, FDR: 0.0012; dorsal back vs. perianal region, FDR: 0019; axilla vs. perianal region, FDR: 0.0091; abdomen vs. perianal region, FDR: 0.0014; perianal region vs. interdigital area, FDR: 0.0125). Results of comparison of other bacterial genera and *p*-values are listed in Supplementary Table 7 in Supplementary material S1.

Among the most abundant 10 genera, significant differences corrected using FDR were observed in *Blautia* (chin vs. perianal region, FDR: 0.0009; nose vs. perianal region, FDR: 0.0348; inner pinna vs. perianal region, FDR: 0.0135; dorsal back vs. perianal region, FDR: 0.0306; axilla vs. perianal region, FDR: 0.0297), *Catenibacterium* (chin vs. perianal region, FDR: 0.0060; nose vs. perianal region, FDR: 0.0023; inner pinna vs. perianal region, FDR: 0.0409; dorsal back vs. perianal region, FDR: 0.0465), *Peptoclostridium* (chin vs. perianal region, FDR: 0.0056; nose vs. perianal region, FDR: 0.0365; inner pinna vs. perianal region, FDR: 0.0091; dorsal back vs. perianal region, FDR: 0.0306; abdomen vs. perianal region, FDR: 0.0495; perianal region vs. interdigital areal, FDR: 0.0324), *Corynebacterium* (chin vs. nose, FDR: 0.0033; chin vs. inner pinna, FDR: 0.0006; chin vs. dorsal back, FDR: 0.0087; chin vs. axilla, FDR: 0.0025; chin vs. abdomen, FDR: 0.0059; chin vs. perianal region, FDR: 0.0124; chin vs. interdigital areal, FDR: 0.0173), *Curtobacterium* (chin vs. inner pinna, FDR: 0.0021; inner pinna vs. perianal region, FDR: 0.0234), *Sphingomonas* (nose vs. axilla, FDR: 0.0391; nose vs. interdigital area: 0.0337). The most abundant genus *Streptococcus* did not have significantly different abundances among eight body sites. Results of comparison of other bacterial genera and *p*-values are listed in Supplementary Table 8 in Supplementary material S1.

**Figure 2:**
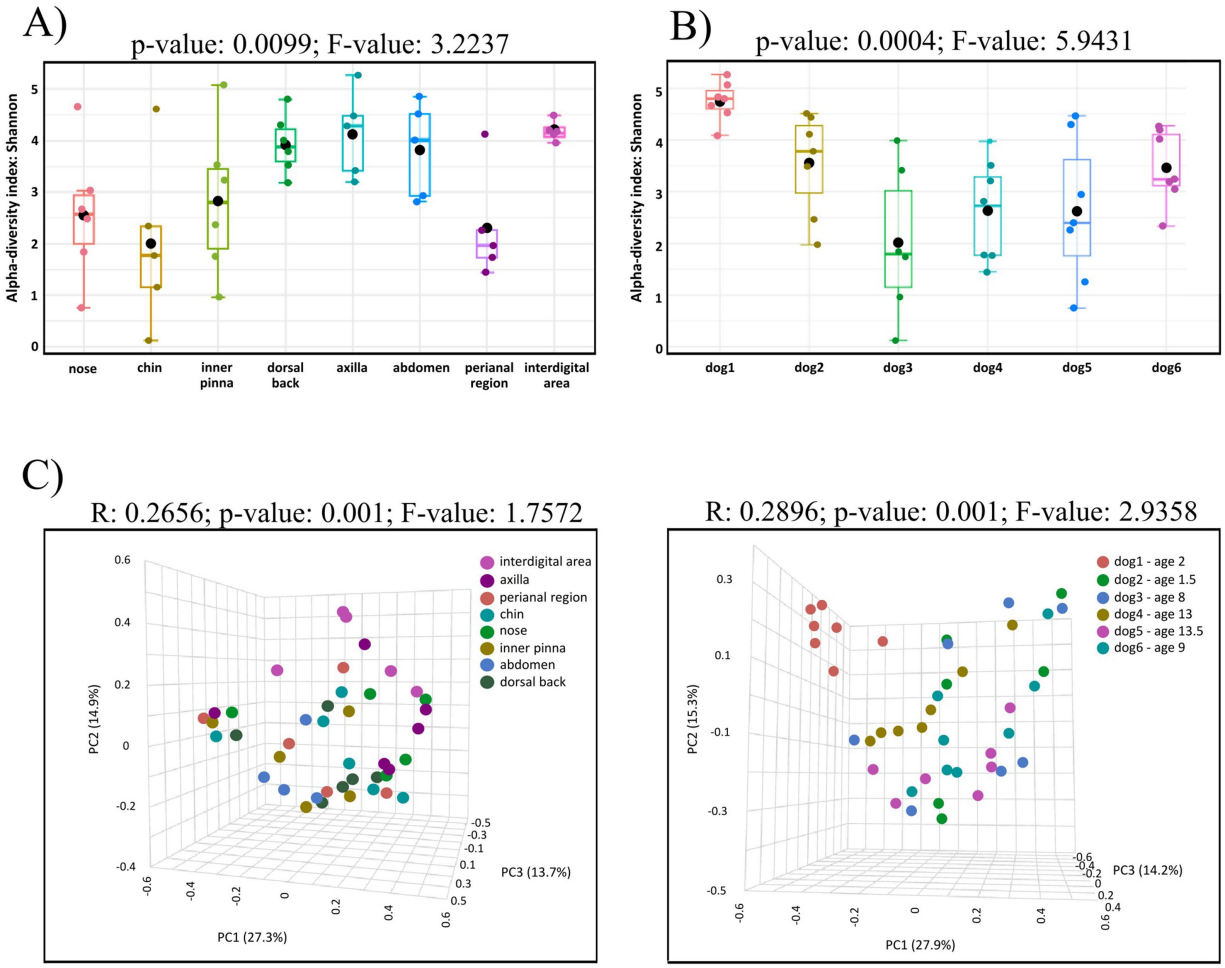
**(A)** Box-plot of alpha diversity indices (Shannon) for the eight studied body sites of healthy dogs. Alpha analysis showed that bacterial diversity differs on individual skin sites (*p*-value: 0.0099, *F*-value: 3.2237) with maximum on the interdigital area and minimum on the chin. **(B)** Box-plots of alpha diversity indices (Shannon) of the skin microbiota of six healthy dogs. Significant differences were seen among individual dogs (*p*-value: 0.0004; *F*-value: 5.9431). **(C)** Beta diversity: community structure profiling of bacterial communities across samples collected from eight skin sites (left) and across skin samples from individual dogs (right) are shown on 2D PCoA plots. Significant differences among groups are observed in both cases. The higher *R*-value on right plot indicates greater differences in genus composition among the studied groups.

Alpha analysis showed that bacterial diversity differs on individual skin sites (*p*-value: 0.0099, *F*-value: 3.2237) with maximum on the interdigital area and minimum on the chin (*p*-value: 0.0416, Figure 2A). Significant differences were seen also between perianal region and dorsal back (*p*-value: 0.0194), perianal region and axilla (*p*-value: 0.0161), perianal region and abdomen (*p*-value: 0.0417), nasal and interdigital area (*p*-value: 0.0274), nose and dorsal back (*p*-value: 0.0489), nose and axilla (*p*-value: 0.0403), chin and interdigital area (*p*-value: 0.0416). However, no significant differences corrected using FDR were detected. The results of all comparisons are included in Supplementary Table 9 in Supplementary material S1. Comparison of bacterial composition among individual dogs showed also significant differences (*p*-value: 0.0004; *F*-value: 5.9431; Figure 2B).

Skin site as an experimental factor and the source of variation in the dataset showed that the results on the PCoA plot revealed eight partially overlapping clusters (*R*: 0.2656; *p*-value: 0.001; *F*-value: 1.7572, Figure 2C left). The variations at the 1st, 2nd, 3rd, axes, were 27.3, 14.9 and 13.7%, respectively. The used pairwise PERMANOVA showed significant differences between these groups of samples: perianal region vs. dorsal back (*p*-value: 0.0010), perianal region vs. inner pinna (*p*-value: 0.0110), perianal region vs. axilla (*p*-value: 0.0080), perianal region vs. interdigital area (*p*-value: 0.0070), nose vs. interdigital area (*p*-value: 0.0310), dorsal back vs. chin (*p*-value: 0.0240). Significant differences corrected using FDR were detected in perianal region vs. dorsal back (FDR: 0.028). The results of all comparisons are included in Supplementary Table 10 in Supplementary material S1.

Another experimental factor–individual dogs also showed significant differences (*R*: 0.2896; *p*-value: 0.001; *F*-value: 2.9358; Figure 2C right). The variations at the 1st, 2nd, 3rd, axes, were 27.9, 15.3 and 14.2%, respectively.

### 3.2 Bacterial skin composition of dogs with inflammatory skin lesions

**Figure 3:**
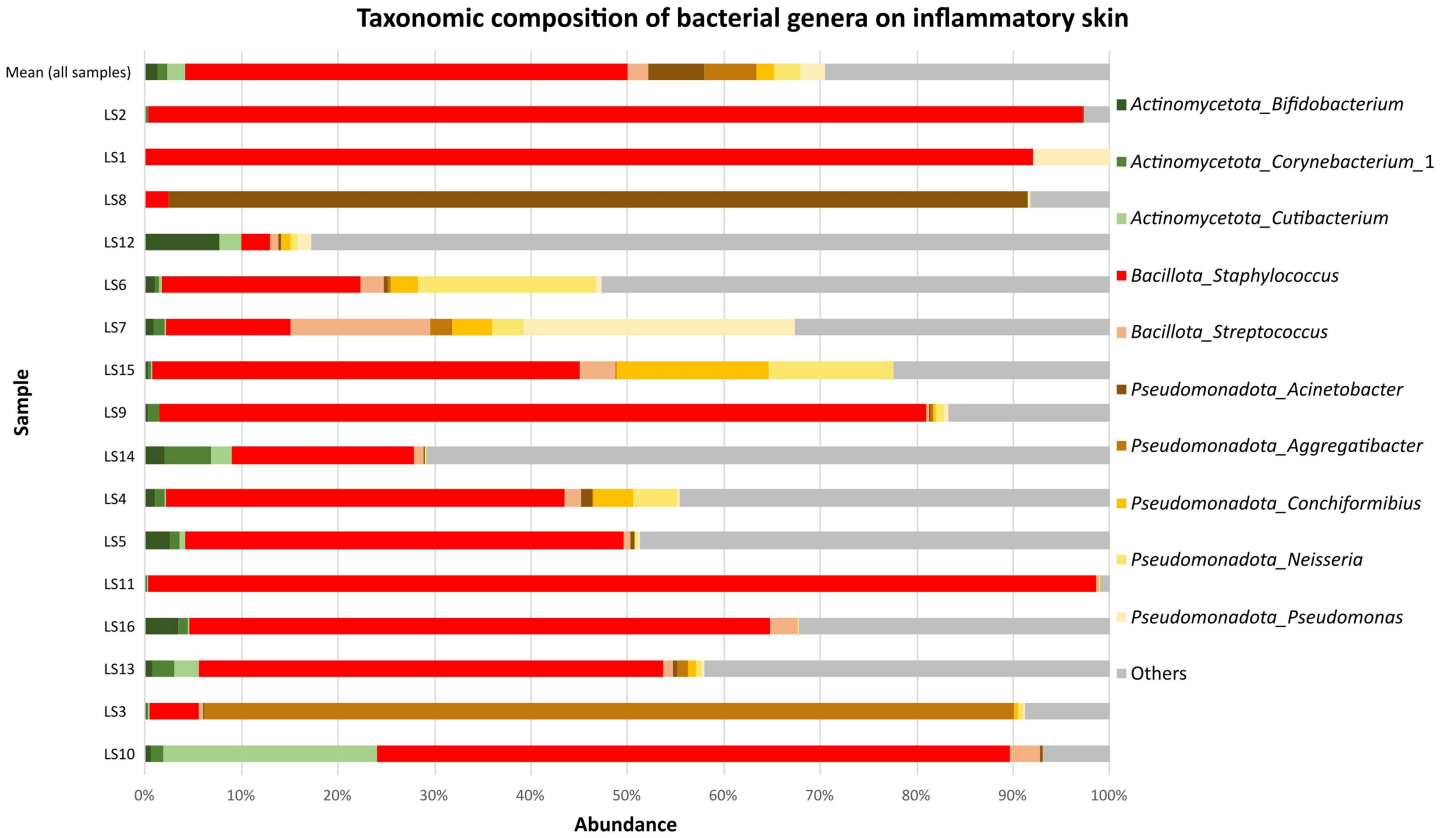
Mean relative abundance of top 10 the most abundant bacterial genera on inflammatory skin (16 samples).

A total of 23 phyla were identified in the samples from patients with inflammatory skin lesions. The microbiota of skin lesions was formed predominantly by the phylum Bacillota (52.8 ± 29.4%). This was followed by Pseudomonadota (26.3 ± 27.6%), Actinomycetota (11.8 ± 11.9%) and Bacteroidota (4.3 ± 4.0%). At genera level, *Staphylococcus* (45.9 ± 32.7%) was dominated at parts of skin lesions compared to healthy skin. Specifically, *Staphylococcus* spp. was the most abundant genus in 12/16 samples collected from diseased skin (Figure 3). Among further abundant genera belonged *Acinetobacter* (5.8 ± 21.5%), *Aggregatibacter* (5.5 ± 20.3%), *Neisseria* (2.7 ± 5.2%), *Streptococcus* (2.1 ± 3.4%) and *Conchiformibius* (1.8 ± 3.9%). Mean relative abundance of other taxa on the parts of skin lesions are summarized in Supplementary Tables 1–3 in Supplementary material S2. Analysis of the core microbiome showed a predominance of *Staphylococcus* (1.00). The rest had prevalence below 0.38 (Supplementary Table 4 in Supplementary material S2).

### 3.3 Comparison of healthy canine skin and skin of dogs with inflammatory skin lesions

**Figure 4:**
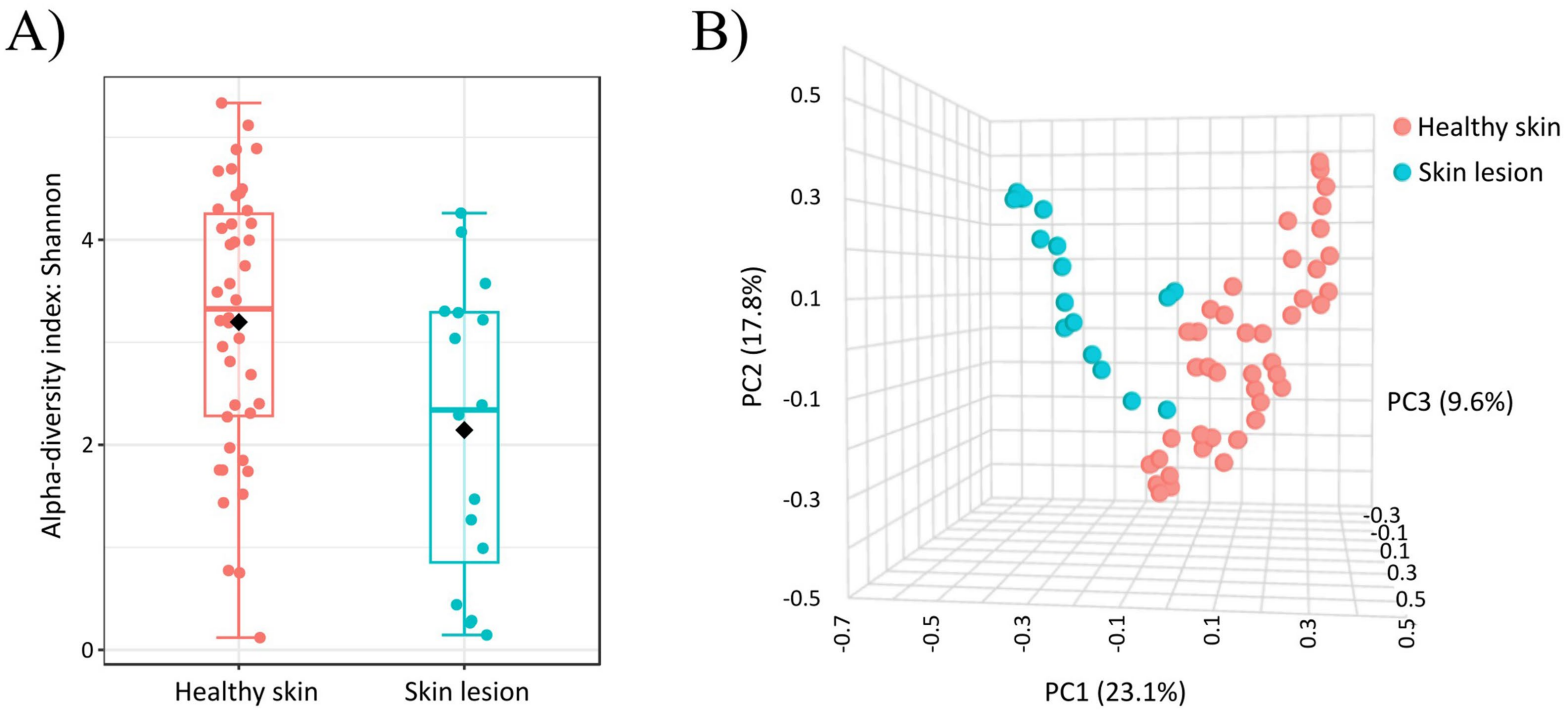
**(A)** Alpha diversity analysis (Shannon index) of bacterial communities on healthy and inflammatory skin. Significant differences between groups were observed for Shannon (*p*-value: 0.0176). **(B)** Beta diversity: community structure profiling of bacterial communities across samples collected from healthy skin and across skin samples from inflammatory skin lesions (2D PCoA plot). The *R*-value indicates differences in genus composition among the studied groups.

The abundance of *Staphylococcus* spp. was significantly higher (*p*-value < 0.0001; FDR: 0.0077) on skin lesions compared to healthy canine skin. In contrast, healthy dogs exhibited a higher abundance of the many other genera, such as *Streptococcus* (*p*-value < 0.0001; FDR: 0.0077), *Curtobacterium* (*p*-value: 0.0072; FDR: 0.1192) or *Micrococcus* (*p*-value < 0.001; FDR: 0.0004). All genera which were significantly different between healthy and unhealthy dogs are shown in Supplementary Table 5 in Supplementary material S2. After comparison the indices of alpha diversity within bacterial populations for healthy skin and unhealthy skin, significant differences were observed for Shannon (*p*-value: 0.0176; Figure 4A). While choosing sample type (healthy or skin with lesions) as an experimental factor the results on a PCoA plot revealed two dominant non-overlapping clusters (*R*: 0.1770; *p*-value < 0.001; Figure 4B). The variations at the 1st, 2, 3rd, axes, were 23.1, 17.8 and 9.6%, respectively. Using the PICRUSt program to analyze the functional profiles of healthy and lesioned skin, we identified 11 predicted metabolic functions at the second level of the KEGG hierarchy. However, no significant differences were observed between the functional profiles of healthy skin and lesion site samples (Supplementary Table 6 in Supplementary material S2).

## 4 Discussion

The use of next-generation sequencing (NGS) techniques to characterize microbial communities has increased in recent years, opening the possibility of readily identifying bacteria that may be not identified by culture-based methods (Abayasekara et al., 2017). These culture-independent studies have revealed that the skin is colonized with a larger number of microbes than previously thought (Hoffmann, 2017). The existence of skin microbiota is of doubtless physiological importance, because these microorganisms play important role in the regulation of delicate immune response of the skin. Additionally, depending on their abundance and composition could be clinically relevant (Carmona-Cruz et al., 2022).

In the first part of the presented study, we focused on the skin of healthy dogs and compared differences on individual skin sites. Generally, we found out that the most abundant phyla were: Bacillota, Actinomycetota, Pseudomonadota and Bacteroidota. According to several studies, these four bacterial phyla are involved in the colonization of human and animal skin, and differences are observed mainly in the abundance of individual phyla (Grice et al., 2009; Cuscó et al., 2017; Older et al., 2017; Apostolopoulos et al., 2021). Recent exploratory study (Apostolopoulos et al., 2021) with 12 healthy dogs found that healthy canine skin was predominantly colonized by Actinomycetota, followed by Pseudomonadota, Bacillota and Bacteroidota. In the aforementioned study, skin swabs were collected from axilla, interdigital area, groin and ear canal so these findings partially agree with our results whereas we detected Actinomycetota as the predominant phylum in the axilla, interdigital area and inner pinna (we did not take swabs from groin).

However, we could observe more variability between studies when the bacterial composition at the genera level is considered. Apostolopoulos et al. (2021) detected that healthy skin of dogs is colonized mainly by *Macrococcus* and *Staphylococus* (> 4%). On the contrary, in our set of healthy dogs we identified these two genera only in very low abundance. In another study, analysis of samples from skin of healthy dogs showed that the most abundant genus was *Ralstonia* spp. (Hoffmann et al., 2014). It is possible that the *Ralstonia* spp. identified in the study were obtained from the environment, due to the dog's frequent interactions with its external environment (Hoffmann et al., 2014). It is very likely since they are gram-negative bacteria, which are primarily considered as environmental microorganisms found in water, soil and plants (Ryan and Adley, 2014). A more recent study showed that the predominant bacteria on healthy canine skin (axilla, pinna, groin) are *Porphyromonas*, *Staphylococcus*, *Streptococcus*, *Propionibacterium* and *Corynebacterium* species (Bradley et al., 2016). Similarly, Chermprapai et al. (2019) documented that the most dominant bacterial taxa shared between four body sites on healthy skin are *Pseudomonas*, *Kocuria*, *Porphyromonas*, *Staphylococcus* and *Corynebacterium*. Our findings partially agree with these results whereas we identified some of these genera also in our study but in much lower abundance (*Corynebacterium_*1: 4.1%; *Staphylococcus*: 2.0%; *Kocuria*: 1.7%; *Propionibacterium*: 1.0%; *Pseudomonas*: 1.4%).

In contrast to other research results, we identified the genus *Streptococcus* in the highest abundance, ranging from an average of 4.4% the total taxa identified in the axilla to 49.0% of the taxa identified in the chin. In addition, our previous study detected the microbiome of healthy canine skin using culture-based methods combined with MALDI-TOF spectrometry identification. Results showed presence of streptococcal strains only rarely (Štempelová et al., 2022) what could be caused by poor growth of streptococci on used culture media. Therefore, current information on the distribution of *Streptococcus* spp. on healthy canine skin is limited. Until now, studies showed that commensal streptococci are presented mainly in the oral and nasopharyngeal microbiota of healthy humans (Oh et al., 2015) and less is known about the role of streptococci on the skin. It was found that streptococci have ability to produce proteases probably involved in the defensive mechanisms of the skin. However, the expression and functions of these proteases, especially those secreted by the common streptococcal skin commensals (*Streptococcus mitis*, *Streptococcus oralis*, and *Streptococcus sanguinis*), have yet to be investigated in detail (Chua et al., 2022). Moreover, dogs appear to be the primary hosts of *Streptococcus canis*. Clinical manifestations of *S. canis* infection range from mild superficial inflammation to severe invasive disease in dogs, cats and humans (Pagnossin et al., 2022).

*Curtobacterium* was the second most abundant genus across all samples taken from healthy canine skin. This genus was the most abundant in the area of inner pinna (significantly more abundant on the inner pinna compared to the perianal area; and inner pinna and chin). This bacterium is closely associated with plants (Evseev et al., 2022), so, similarly like *Ralstonia* spp., it may be related to the natural behavior of dogs and their habit of being in close contact with their environment. We also detected the genus *Bacteroides* in high abundance. This genus was also identified on canine skin of 12 healthy dogs of different breeds (Hoffmann et al., 2014), but it was also found in higher abundance in patients with acne vulgaris (Kelhälä et al., 2018).

After that, we could observe significant variations among some genera and location of the skin confirmed by the use of FDR. The most common, significant variations were observed in genera such as *Massilia*, *Deinococcus*, *Rubellimicrobium* or *Brevundimonas*. Similarly, significant variations were documented also in *Curtobacterium*, *Peptoclostridium* or *Sphingomonas*. However, the most abundant genera *Streptococcus* and *Bacteroides* did not differ significantly on different parts of the body.

Studies have also looked at how diverse skin microbiota is. Hoffmann et al. (2014) investigated microbiota diversity among skin sites. They observed higher species richness in the samples from haired skin when compared to mucosal surfaces or mucocutaneous junctions. In the same way, another study showed that mucocutaneous perianal region and nasal skin have lower alpha diversity values when compared to all other haired skin regions (Cuscó et al., 2017). These results are consistent with our current observation whereas we found some variances in bacterial diversity on individual parts of the body. The species evenness (Shannon) was higher on axilla, interdigital area, abdomen and dorsal back. The species evenness (Shannon) significantly differs in pairwise comparisons between perianal region vs. axilla, perianal region vs. interdigital area and perianal region vs. dorsal back.

The biological significance of alpha diversity is caused by complex interaction among microclimatic conditions (temperature, humidity, air exposure), physiological conditions on the canine skin (coat density, skin folds, distribution of sebaceous and sweat glands), dog behavior and environmental factors. In our study, higher alpha diversity in the interdigital region compared to the chin may be due to several factors. The chin is often affected by microorganisms from the dog's saliva and oral cavity, which can be dominant and reduce the diversity of other microorganisms. Additionally, a dog's saliva saliva may contain antimicrobial peptides and enzymes (lysozyme) that can inhibit the growth of certain types of bacteria. The interdigital area is characterized by the presence of eccrine sweat glands, which increase the humidity of the area and support the presence of a diverse community of microorganisms. In addition, mechanical friction in the interdigital area can cause a dynamic exchange of microorganisms, which increases diversity. We also observed a higher diversity in the area of the back and abdomen. It may be due to the fact that the conditions on the back and abdomen are relatively neutral - they are not too wet or too dry (moderate number of sebaceous glands). These areas are also less exposed to mechanical stress. The microbial community in the perianal region can be made up of bacteria that originate from the digestive tract (less diverse). It is also an area with sebaceous glands. And at the same time, this area is not exposed to the outside environment. The result of these factors is low bacterial diversity in our study. In addition, we observed that the species evenness (Shannon) significantly differs also among individual dogs.

Furthermore, we focused on beta diversity analysis to express the differences among samples. Results of beta-diversity showed the fact that the main force driving the skin microbiota composition is the individual, followed by the skin site (based on *F*-values). Cuscó et al. (2017) focused on the skin microbiota in homogenous cohort of dogs. They suggest that the main force driving the skin microbiota composition is the individual even in dogs cohabiting and interacting together.

Bacteria play important role in both, health and disease and changes in bacterial community composition are associated with many skin diseases (Hoffmann, 2017; Karkman et al., 2017). In the next part of the research, we focused on the skin microbiome in dogs with dermatological disease because overall role of the skin microbiome in diseases is poorly understood, especially in canine skin. In our study, samples obtained from inflammatory skin lesions were dominated by *Staphylococcus* spp. Although staphylococci may have protective abilities (production of antimicrobial peptides), it is likely that their increase in dermatological diseases is a consequence of their pathogenic properties. Species such as *Staphylococcus pseudintermedius* (Horsman et al., 2025) or *Staphylococcus aureus* (Costa et al., 2022) are often caught in dogs at the site of skin lesions. They produce toxins, enzymes and factors that disrupt the skin barrier, increase inflammation and promote infection (Tam and Torres, 2019; Carroll et al., 2021). As a result, the balance between “good” and “bad” bacteria is disturbed leading to the dominance of pathogenic species.

Increased abundances of staphylococci have been observed in skin diseases such as pyoderma (Older et al., 2020) or atopic dermatitis in both humans and dogs (Kong et al., 2012; Bradley et al., 2016). However, increase in abundance of staphylococci in allergic dogs was not significant in another study (Hoffmann et al., 2014). Apostolopoulos et al. (2021) showed that skin microbiota of allergic dogs is created by *Sphingomonas*, *Staphylococcus*, *Clostridium sensu stricto_* 7, and *Nocardioides* (Apostolopoulos et al., 2021). In contrast to these findings, we captured these genera in ill dogs only in lower abundance.

Subsequently, we compared samples obtained from healthy skin and from the skin of dogs with dermatological disease. Higher abundance of genera such as *Streptococcus*, *Curtobacterium*, *Micrococcus* or *Macrococcus* were observed on healthy canine skin. On the contrary, the genus *Staphylococcus* was markedly predominant in samples from skin lesions. Interestingly, Apostolopoulos et al. (2021) found that *Sphingomonas* and *Nocardioides* were significantly higher abundant in allergic compared to healthy dogs. In the same study, lower species richness was observed in allergic dogs. Similarly, our results showed that alpha diversity was lower on skin lesions compared to healthy canine skin. In beta diversity analysis, clustering was detected across samples from healthy skin and skin lesions what indicates higher similarity among healthy skin samples and samples from skin lesions.

In conclusion, we found taxonomic differences between healthy canine skin and skin lesions. *Streptococcus* spp. was the predominant bacterial genus on healthy skin of dogs whereas skin lesions were predominantly colonized by the genus *Staphylococcus*. Variances in bacterial diversity on individual parts of the body were observed. Alpha diversity analysis (Shannon index) showed maximum on interdigital area but minimum on a chin. Beta analysis showed clustering across samples from individual dogs, but also among samples from individual parts of skin. Variability was observed among samples from healthy and unhealthy skin.

## Data Availability

Illumina MiSeq sequencing data are available in BioProject SRA database under accession number PRJNA1143188 (accession numbers are available in Supplementary Table 7 in Supplementary material S2).
